# Variations in Key Aroma Compounds and Aroma Profiles in Yellow and White Cultivars of *Flammulina filiformis* Based on Gas Chromatography–Mass Spectrometry–Olfactometry, Aroma Recombination, and Omission Experiments Coupled with Odor Threshold Concentrations

**DOI:** 10.3390/foods13050684

**Published:** 2024-02-23

**Authors:** Wei Song, Min Sun, Huan Lu, Shengyou Wang, Ruijuan Wang, Xiaodong Shang, Tao Feng

**Affiliations:** 1 School of Perfume and Aroma Technology, Shanghai Institute of Technology, Shanghai 201418, China; 2 Institute of Edible Fungi, Shanghai Academy of Agricultural Sciences, Shanghai 201403, China; 3 Institute of Edible Fungi, Sanming Academy of Agricultural Sciences, Sanming 365000, China; 4 Fujian Key Laboratory of Crop Genetic Improvement and Innovative Utilization for Mountain Area, Sanming 365509, China

**Keywords:** *Flammulina filiformis*, key aroma compounds, aroma recombination and omission, aroma profiles difference, odor activity value

## Abstract

*Flammulina filiformis* (*F. filiformis*) is called the ‘benefiting intelligence’ mushroom. There is a notable difference between a yellow cultivar (with a robust aroma) and a white mutant cultivar (with a high yield) of *F. filiformis*. A thorough analysis of aroma differences is essential to improve the aroma of high-yield strains. This study employed a combination of gas chromatography–mass spectrometry–olfactometry (GC-MS-O) and aroma extract dilution analysis (AEDA) to analyze the variations in aroma compounds. Then, the contribution of the odorants was determined using flavor dilution (FD) factors and odor activity values (OAVs). Aroma omission and recombination experiments were used to identify the key odorants. A total of 16 key aroma compounds were characterized in *F. filiformis*, along with four eight-carbon volatiles (3-octanone, 3-octanol, octanal, and 1-octen-3-ol). Finally, the dominant aroma characteristic was “sweet” for the yellow strain, while it was “green” for the white strain. More research is required to investigate the enzymes and corresponding genes that regulate the synthesis of aroma compounds in *F. filiformis* for future breeding programs.

## 1. Introduction

*Flammulina filiformis* (*F. filiformis*), or East Asian needle mushroom, falls under the family *Physalacriaceae* and the order *Agaricales* [1,2]. *F. filiformis* is a highly valued edible fungus due to its high nutritional and medicinal value. Additionally, it is one of the most productive edible types of fungus in industrialized cultivation [2]. There are two distinct strains of *F. filiformis*, identified by their stipe color in both yellow and white forms [3]. Most of the *F. filiformis* currently consumed in the Asian market consists of the white strains with high yield and the yellow strains with a robust aroma [2]. Therefore, to generate a strain of *F. filiformis* that exhibits high yield and a robust aroma, an in-depth analysis of *F. filiformis* with desirable traits is crucial for breeding.

In addition, aroma is a crucial quality in edible fungi, and various aroma compounds have been characterized in *F. filiformis* in previous studies [4,5,6,7,8]. Eight-carbon compounds, including 3-octanol and 3-octanone with green and mushroom notes, contribute significantly to the aroma of *F. filiformis* [9]. Interestingly, *F. filiformis* volatiles were significantly dynamic in various cultivars [10]. Conversely, few studies have reported on the differences in aroma profiles between yellow and white cultivars of *F. filiformis*.

Furthermore, a thorough analysis of the differences in key aroma compounds is essential to improving the aroma of white mutant cultivars. Calculating the odor activity values (OAVs) is a crucial method utilized to identify key odorants. However, current research on OAVs exhibits certain limitations, particularly with regard to odor thresholds [11,12,13]. Most odor thresholds for aroma compounds were obtained from literature searches, resulting in inaccurate overall aroma profiles due to a lack of knowledge on the aroma contribution of some odorants (not available in the literature) [14]. Accurately measuring the threshold of key aroma compounds could help obtain richer and more complete aroma profiles.

In summary, this study aimed to analyze the differences in aroma profiles between yellow and white *F. filiformis*. Odorants were identified and quantified using gas chromatography–mass spectrometry–olfactometry (GC-MS-O) and external standard methods. Later, based on flavor dilution factors (FDs) and OAVs, key aroma compounds were determined and their contributions confirmed through aroma recombination and omission experiments. Through these experiments, an aroma profile for *F. filiformis* was established. Finally, the results will offer important insights into a comprehensive characterization of the aroma profile of *F. filiformis*, along with a better comprehension of the aroma variations between yellow and white cultivars.

## 2. Materials and Methods

### 2.1. Mushroom Samples and Chemicals

Three cultivars of *F. filiformis* that differ in colors, caps, and stipes were provided by the Institute of Edible Fungi (Shanghai Academy of Agricultural Sciences, Shanghai, China) and were named F1, F2, and F3, respectively. At their mature stage, three cultivars, with distinct features as outlined in Table S1, were identified by a stipe color range that spans from light yellow (F1) to yellow (F2) and white (F3) [15]. Through the crossbreeding of F1 and F3, F2 was developed. The *F. filiformis* samples were kept on ice during transportation, frozen using liquid nitrogen, and then pulverized into a fine powder using a blender (Shanghai Wansheng Co. Ltd., Shanghai, China). The powder samples were properly packaged and stored at −20 °C for further use. Commercially, the authentic standards were available (listed in Supplementary Materials). All chemicals used were analytical grade or higher.

### 2.2. Headspace Solid-Phase Microextraction (HS–SPME) Analysis

Based on a previously reported method with a minor alteration, preliminary experiments were conducted to optimize the HS-SPME procedures [16]. Each *F. filiformis* sample (5 g) was weighed into a 20 mL headspace vial, followed by the addition of saturated saline (3 mL) and 1,2-dichlorobenzene (2 μL, 100 mg/kg, solvent: acetone). The samples were equilibrated for 3 min in a water bath at 55 °C. A flexible fiber coated with a 50/30 μm layer of PDMS/DVB (Supelco, Bellefonte, PA, USA) was utilized to extract the volatile compounds at 55 °C for 50 min. Subsequently, the SPME fiber was removed and promptly placed into a GC injector for desorption at 250 °C (5 min), followed by detection. And the samples were injected in splitless mode.

### 2.3. Solvent-Assisted Flavor Evaporation (SAFE)

At room temperature, samples (30 g) and dichlorobenzene (300 μL) were stirred (3 × 1 h) with dichloromethane (3 × 100 mL). After combining the organic phases, they were dried using anhydrous sodium sulfate and then transferred into a 500 mL distillation flask, which was a part of the SAFE apparatus (Glasbläserei Bahr, Manching, Germany). The SAFE process involves the separation of volatile fractions using relatively low temperatures (40 °C) and high vacuum pressures (5 × 10^−5^ mbar). The distillate was concentrated using a nitrogen stream until the final volume was 1 mL. For GC-MS, 2 μL of concentrate was injected into the injection port at 250 °C.

### 2.4. Gas Chromatography–Mass Spectrometry–Olfactometry (GC-MS-O) Analysis

The experiment employed a gas chromatography 8860 system coupled with a 5977B mass spectrometer (Agilent Technologies, Santa Clara, CA, USA). An HP-INNOWAX analytical fused silica capillary column (60 m × 0.25 mm × 0.25 μm, Agilent Technologies, USA) was used to isolate volatiles. The oven heating procedure involved programming at 40 °C (maintained for 3 min) and ramping at 5 °C/min to 100 °C (held for 5 min), followed by an increase to 210 °C (held for 5 min) at 3 °C/min. Helium (>99.99%) was used as the carrier gas, maintaining a constant flow at 1.0 mL/min in splitless mode. The mass spectrometer was configured to use an electron ionization mode at a 70 eV ionization energy and a 230 °C ion source temperature. It was scanned for a range of 30 to 450 *m*/*z* in full scan mode.

The GC-O experiment utilized an Agilent 8860 gas chromatography system that was equipped with an olfactory detection port (Gerstel, Mülheim an der Ruhr, Germany; ODP-4). The chromatographic conditions were consistent with the GC-MS method, and the flow split ratio was 1:1 between the detector and the olfactory port at the end of the column. To reduce nasal discomfort and fatigue, the GC-O evaluation sessions were divided based on elution time into 2 segments: 0–31 and 32–62 min (recording of compounds, aroma characteristics, and retention times was carried out by at least 3 assessors).

### 2.5. Aroma Profile Analysis

The aroma profiles were measured at the Sensory Laboratory located in the Shanghai Institute of Technology (Shanghai, China), following the International Standard Guidelines (ISO) 8589-2007 [17]. The Ethics Committee at the Shanghai Institute of Technology approved the sensory analysis. The sensory panel comprised 10 sensory assessors (5 males and 5 females aged from 22 to 30 years old, all of whom signed informed consent forms) following ISO 8586-2023 for selection, training, and monitoring [18]. Once acquainted with the *F. filiformis* aroma, the assessors were requested to articulate and identify aroma descriptors by discussing aroma attributes [12]. The 7 aroma descriptors, which include sweet (*δ*-dodecalactone), fatty (decanol), cheese (nonanoic acid), mushroom (1-octen-3-ol), floral (terpineol), green (2-penten-1-ol), and fruity (ethyl 3-hexenoate), were identified along with their reference standards through discussion among the sensory panel. The aroma intensities (AIs) were evaluated using a 10-point scale, following a previously reported method with minor changes, ranging from 0 to 9, wherein “0”, “1”, “5”, and “9” correspond to none, weak, moderate, and strong. All sensory evaluations were conducted by this sensory panel in triplicate.

### 2.6. Aroma Extract Dilution Analysis (AEDA)

For the AEDA, the *F. filiformis* extract samples underwent stepwise dilution in a 1:2 ratio with dichloromethane solvent before being injected for sniffing until no odorant could be detected [13]. In addition, if the volatile extract was acquired using SPME, it was diluted by gradually adjusting the split ratio to 1:2. The dilution was determined by the flavor dilution (FD) factor (indicated by the odorant maximum dilution).

### 2.7. Identification and Quantification of the Key Aroma Compounds

Volatile compounds were identified based on odor characteristics using an authentic method by comparing retention indices (RIs) with reference standards (https://webbook.nist.gov/chemistry/, accessed on 7 August 2022) and mass spectra with data from the NIST Mass Spectrometry Data Center (2023 version) [19].

The aroma-active compounds were quantified by constructing external standard curves. For the matrix preparation, dichloromethane (300 mL) added to F2 (30.0 g) was extracted for 12 h. The extraction was repeated until nothing was detected by SAFE-GC-O-MS. The aroma-active compound standards at different concentrations (0.004–0.149 mg/kg) were mixed with 1,2-dichlorobenzene (300 μL, 100 mg/kg), then diluted in the matrix prepared above at 7 different concentration ratios (1:5, 1:10, 1:50, 1:100, 1:200, 1:400, and 1:1000). Subsequently, the above solution mixture was extracted using the SAFE or HS-SPME method and analyzed through the GC-MS procedure (described in Section 2.4), with the exception that MS was performed in selected ion monitoring (SIM) mode [20].

To construct the calibration curves, the ratio between the peak area of the quantified compounds and that of the internal standard (1,2-dichlorobenzene) was plotted against the ratio of their respective concentrations (listed in Supplementary Materials). All analyses were conducted three times to ensure accuracy. The limits of detection (LODs) referred to the concentration of a standard compound whose signal-to-noise (S/N) ratio was 3, while the limit of quantitation (LOQ) was 10 [21]. LOD and LOQ data are listed in Table S4.

### 2.8. Odor Threshold Concentrations

Odor threshold concentrations were measured using the three-alternative forced choice (3-AFC) test in the mushroom matrix (matrix preparation mentioned in Section 2.7) based on ASTM E679-19 (ASTM, 2019) [22]. Seven concentration groups were established for testing, and each group comprised a spiked sample and two matrix samples. The concentrations of the odorants (set with a three-digit random code) were decreased in turn with a dilution factor of three and presented to panelists in ascending order [23]. Supplementary Table S2 contains the maximum concentrations utilized for each compound. Before determining the OT, the panelists were presented with a spiked reference sample (with a concentration equivalent to the fourth level) to showcase the respective aroma characteristics. Samples (10 mL) were placed in transparent vials covered with plastic lids at room temperature (22 ± 1 °C). A one-minute break was imposed between each test, and each OT test was carried out three times in a row.

The statistical analysis was performed on all the results obtained from the 3-AFC test. OTs for each odorant were calculated using the S-curve fitting (CF) method. Further corrections were implemented using the following formula:

(1) P=3×p−12

where *P* represents the correction value for the correct recognition probability, and *p* represents the measured correct identification probability value (the percentage of panelists who correctly identified the spiked sample). Logarithmic concentration and detection probability curves were created for 7 different concentration points, with the log (concentration) plotted on the X-axis. The S-curve equation was ultimately utilized for fitting, and the resulting formula was as follows [24]:

(2) $$P=\frac{1}{1+e^{\frac{x-C}{D}}}$$

where *P* represents the correction value for the correct recognition probability. *C* and *x* are used to represent the logarithmic concentration and OT, respectively, and *D* represents the characteristic parameter for the odorant, which relates to the gradient for the function [25].

### 2.9. Odor Activity Values (OAVs)

The OAV was used to assess the contributions of the aroma compounds to the overall aroma of *F. filiformis*, which was determined by calculating the ratio of their concentrations to the odor detection threshold.

### 2.10. Aroma Omission/Recombination Experiments

The aroma omission and recombination experiments were conducted as previously described with a slight modification [19]. To verify the individual or group contribution of odorants (OAV ≥ 1) to the overall aroma of *F. filiformis*, omission models were created by omitting a single or a group of specific aroma-active compounds from the complete reconstituted model (Table 3). Furthermore, triangle tests (ISO 4120, 2021) [26] were carried out by 10 panelists to compare the odor differences between the omission models and the complete recombinant models [26,27]. For the omission experiments, significant differences were determined by counting the panelists (a total of 10 individuals) who could recognize the odor differences 0–3 (*p* > 0.05, −), 4–6 (*p* ≤ 0.05, *), and 7–10 (*p* ≤ 0.01, **).

For the recombination experiments, quantified odorants that contribute significantly to the overall aroma (with significant differences validated by panelists in the omission experiment) were mixed in the matrix (mentioned in Section 2.7). Subsequently, the sensory panel (described in Section 2.5) compared the aromas between the recombinant samples and the actual model of *F. filiformis* through triangle tests (ISO 4120, 2021) to construct the final recombination model [26,28].

### 2.11. Statistical Analysis

The statistical analysis was presented as the mean value plus or minus the standard deviation (SD) using Microsoft Office Excel 2016. SPSS 26 (SPSS Inc., Chicago, IL, USA) was used to conduct the Student–Newman–Keuls test to determine the significant differences (*p* < 0.05) among individual samples for each aroma characteristic (we performed a one-way analysis of variance). TBtools version 1.132 [29] was used to create a cluster heatmap of the volatile compounds in *F. filiformis*. The correlations between sensory attributes and aroma-active compounds were analyzed by PLSR using SIMCA version 14.1 (Sartorius Stedim, Umea, Sweden).

## 3. Results and Discussion

### 3.1. Identification of the Aroma-Active Compounds in F. filiformis

A total of 53 volatiles were identified from *F. filiformis* using GC-MS, including 4 acids, 18 alcohols, 7 aldehydes, 5 ketones, 15 esters, and 4 other compounds (Table 1). F1 and F2 (yellow cultivars) were found to contain 27 and 29 compounds, respectively. On the other hand, 15 odorants were detected in F3 (white cultivar). The extraction method is crucial to enhancing the volatile compounds in *F. filiformis*. Previous research has indicated that a combination of two extraction methods can lead to a more effective enrichment of aroma compounds, resulting in an increased quantity and variety [19]. Referring to previous research [19], different pretreatment methods were compared in detail. Table 1 summarizes the volatile data obtained from two pre-treatment methods.

**Figure 1:**
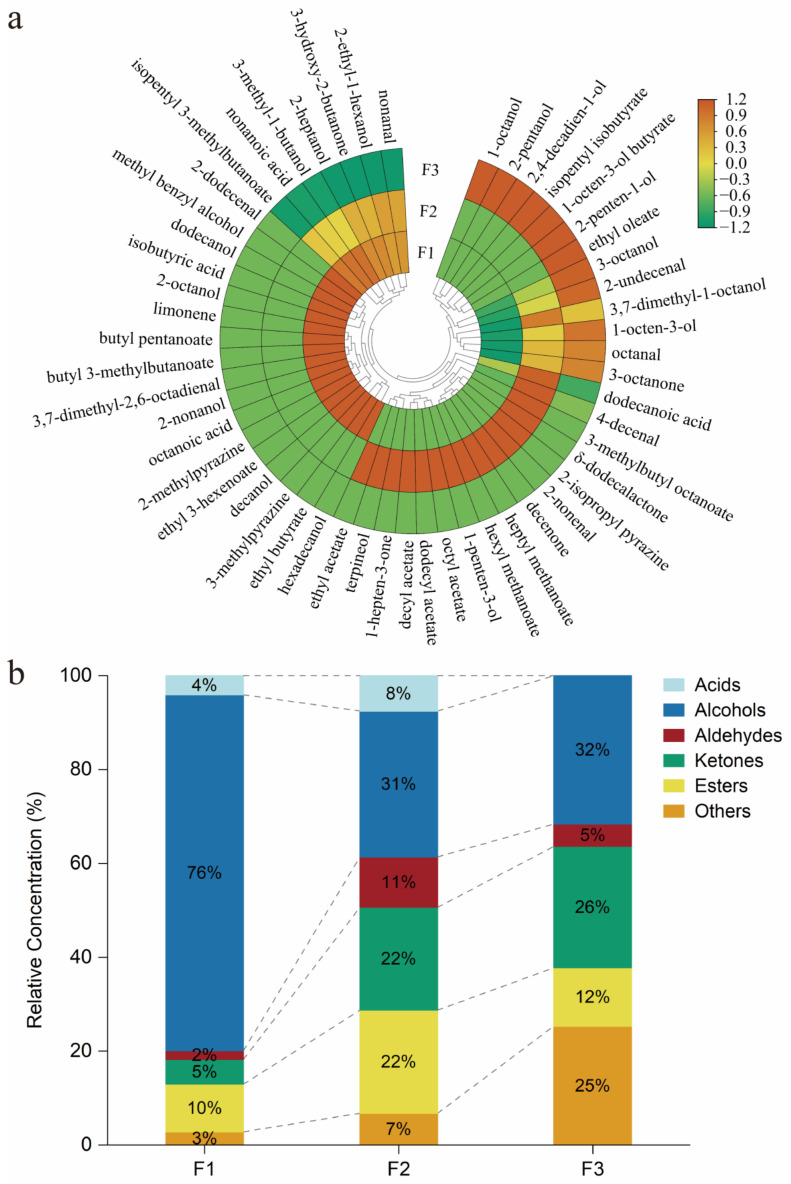
(**a**) Heatmap analysis of the volatile aroma compound contents identified in *F. filiformis* cultivars; (**b**) compositions of volatile compounds in *F. filiformis* cultivars: F1, F2, and F3, respectively.

A visual comparison was conducted using a heatmap analysis to determine the variations in aroma compounds across various *F. filiformis* cultivars (Figure 1a). The color intensity was determined by a normalized scale, with the upper limit set to 1.2 (orange) and the lower limit to −1.2 (green). It is capable of discerning between different cultivars and presenting the relative abundances of volatiles, ranked from highest to lowest. The names of the compounds are mentioned on the outer edge of the fan, while the names of the samples are on its inner edge. The cluster analysis revealed varying cultivars and quantities of distinctive and common compounds present in the concentration results of the three diverse cultivars of *F. filiformis* [10]. To begin with, two volatiles were odorants commonly presented in F1, F2, and F3. 3,7-Dimethyl-1-octanol and octanal belong to eight-carbon compounds linked to typical mushroom notes. Octanal also acted as an info-chemical that inhibits fungal growth and interferes with mycotoxin production [10,30].

Furthermore, F1 contained 27 unique compounds. Interestingly, limonene, also identified in *Volvariella volvacea*, exhibits an enantioselective odor, described as a lemon odor [9,31]. A total of 29 distinct compounds were present in F2. Dodecanol and dodecanoic acid were found in the F1 variety and discovered to be hydroxylated at the ω-7 position to form *δ*-dodecalactone by sub-terminal fatty acid hydroxylases, which is crucial for future research on cultivating and transforming the aroma among different cultivars of *F. filiformis* [32]. F3 comprised 15 distinctive compounds. 1-Octanol (an eight-carbon compound) has been found to inhibit fungal spore germination and is produced through a reduction from 1-octanal, with alcohol dehydrogenase as its source [10,33].

Furthermore, F1 and F2 both contained 10 odorants. Nonanal has been identified as a major aroma compound in various edible mushrooms [9]. 3-Hydroxy-2-butanone, not commonly found in raw mushrooms, has been detected in cooked pine mushrooms [34]. There were eight compounds commonly found in both F2 and F3. They comprised eight-carbon compounds reportedly associated with distinct functions. 3-Octanol served as an inhibitor for both plant growth and seed germination [10]. 1-Octen-3-ol, “mushroom alcohol”, has various functions such as inhibiting fungal growth, promoting seed germination, inducing conidiation, defending plants, and affecting mycotoxin production [10]. It is also possible to identify the origin of the detected 1-octen-3-ol, whether fungal or vegetal, by analyzing its stereochemistry and accompanying compounds [33]. 3-Octanone exhibits inhibitory effects on the fungal spore germination process [10].

The 53 compounds are classified in Figure 1b, wherein it can be observed that the volatiles differ in terms of their types, contents, and amounts among the three cultivars. It was obvious that high overall alcohol concentrations existed among all the cultivars. Alcohols are primarily synthesized by reducing aldehydes through alcohol dehydrogenase. It was visually observed that a higher alcohol content was associated with a lower aldehyde content in the various strains of *F. filiformis*, which is related to alcohol dehydrogenase activity [33]. In the future, clarifying the regulatory genes of aroma compounds in edible mushrooms would be crucial in cultivating cultivars of *F. filiformis* with more appealing scents for consumers [35]. The concentrations of acids, aldehydes, and esters showed a consistent trend, peaking in F2 and having lower values in F1 and F3. Acid compounds, in particular, had 0% content in F3. The content of esters in F2 was higher than in the other two cultivars. Additionally, all three cultivars exhibited an increasing trend in the content of ketones. And other compounds included pyrazines, furans, and sulfur compounds. Overall, the yellow strains contained relatively low levels of aldehydes, particularly in F1, where it was less than 10% of the total content. And esters, ketones, and alcohols were present at relatively high levels in F2, making up more than 70%. Conversely, in the white strains (F3), the content of esters and aldehydes was quite low.

### 3.2. Further Confirmation for Aroma Attributes by GC-O and Contributions by AEDA

Although various volatiles were identified through GC-MS, only a few contributed to the overall aroma, and these are considered to be key aroma compounds in *F. filiformis*. GC-O combined with AEDA was utilized to characterize the primary aroma-active compounds in three cultivars. Then, a sensory evaluation and GC-O were used to determine seven aroma descriptors, including sweet, fatty, cheese, mushroom, floral, green, and fruity notes, for further research.

As shown in Table 1, compared to the white strains, most compounds in the yellow strains have higher FD factors. Out of 38 compounds with an FD ≥ 2, F1, F2, and F3 contained 16, 22, and 6 aroma-active compounds, respectively. In general, most odorants with a high FD (FD ≥ 64) were associated with sweet and typical mushroom notes and were found mainly in yellow strains. It could be further inferred that the sweet notes in the yellow cultivar contributed more to the overall aroma, in comparison to the white cultivar. Yellow strains have been found to contain a variety of sweet note compounds, further classified into distinct types, such as fruity-sweet and honey-sweet. This observation might lay the foundation for exploring the underlying causes of the differences in sweet notes among yellow cultivars.

Compounds with a high FD factor could be responsible for the unique aroma characteristics in samples [36]. Specifically, several sweet note odorants with an FD of 1024 contributed to the sweet aroma in the yellow strains, such as 3-methylbutyl octanoate. The above compounds contributed less to the aroma in the white *F. filiformis*, possibly explaining why such strains lack a sweet aroma sold on the market [1]. Furthermore, 2-penten-1-ol, presented as a green aroma, had the greatest FD factor in the white cultivars, which might potentially lead to a dominant green aroma.

Moreover, the yellow *F. filiformis* had a higher FD factor compared to the white. The distinct mushroom aroma in the yellow variety was caused by these volatiles, including 3-octanol (FD: 512 (yellow) and 1 (white), mushroom), 1-octen-3-ol (FD: 4 (yellow) and 1 (white), mushroom), and 3-octanone (FD: 512 (yellow) and 4 (white), mushroom). Similar key odorants have also been identified in different mushroom cultivars [37]. The variation in aroma in *F. filiformis* could be attributed to these odorants.

### 3.3. Quantitative Analysis and OAV Referring to Volatile Compounds

As shown in Table 2, F1, F2, and F3 contained high concentrations of aroma compounds, such as 3-hydroxy-2-butanone (27.9547 mg/kg, sweet) and 3-octanone (1.6570 mg/kg, sweet). However, the concentration of odorants might not always reflect their contribution to the overall aroma. To accurately assess their aroma contribution, it was necessary to consider their odor thresholds [14]. The odor thresholds for these compounds were measured in the matrix of *F. filiformis* (Table 2). Due to the presence of the largest number of key volatiles, the F2 cultivar was selected to prepare the matrix for odor threshold measurement.

In the realm of food aroma research, compounds with an OAV ≥ 1 were considered key aroma compounds [11]. The OAV of each compound was calculated by dividing the concentration by the odor threshold value, as presented in Table 2. A total of 28 key aroma compounds were identified in the three cultivars. And 14, 19, and 6 key aroma compounds were identified in F1, F2, and F3, respectively. Among these odorants, octanal was present as a common key aroma compound in three cultivars, contributing to fruity notes, respectively. 2-Penten-1-ol was a key aroma compound unique to F3. This could explain the variation in aroma among the three types of *F. filiformis*.

First of all, there were six key volatiles with an OAV > 1000 in F1. Secondly, there were 10 key volatiles with an OAV > 1000 in F2. Furthermore, distinctive key aroma compounds found in F2 might give rise to sweet and mushroom notes. The F2 sweet note was attributed to 3-octanone (sweet), which was in line with previous studies [8]. Likewise, F2 presented a strong fruity note because of the odorant octanal (fruity), with a low threshold (0.0034 mg/L) and concentration (0.0317 mg/kg). On the whole, the key compounds found in the yellow cultivars were mostly characterized by sweet notes. The unique sweet compounds in the two yellow strains could potentially serve as primary factors in distinguishing different strains of *F. filiformis*. Finally, in F3, 3-octanone, octanal, 2-penten-1-ol, 3-octanol, and 1-octen-3-ol were the key aroma compounds with an OAV > 1000. It was apparent that the F3 white strain possessed a noticeable green note, attributed to the presence of 2-penten-1-ol (green).

### 3.4. Aroma Recombination and Omission Experiments

Further aroma recombination and omission experiments were conducted to verify the impact of key aroma compounds in *F. filiformis*. To conduct omission experiments, the triangle test method was employed, and 28 odorants with an OAV ≥ 1 were determined [13]. As shown in Table 3, aroma models were developed to examine the impact of singular or clustered volatile compounds (based on aroma notes) on the overall aroma of *F. filiformis*. These odor models were compared with the omission compounds one by one and with the complete models. In addition, the sensory panel members were required to accurately describe the detected differences in odor [12]. In the sensory panel, 90% of the participants were able to distinguish differences in aroma between the model missing 3-hydroxy-2-butanone (1-2) and the complete model. They noted that the deficient model had a less creamy and sweet aroma as compared to the complete model. The study found that the compounds had low OAVs and were not significant contributors to the aroma of *F. filiformis*. This is consistent with previous studies, which suggested that compounds with low OAVs may not necessarily have a strong impact on the overall aroma [38]. In addition, there are seven aroma note omission models in Table 3. The omission of five aroma attributes, namely sweet, mushroom, floral, green, and fruity, caused the most significant (*p* ≤ 0.01) impact on the overall aroma of *F. filiformis*. On the contrary, the influence of the omission of fatty and cheese notes on the overall aroma was relatively minimal (*p* ≤ 0.05) due to the lesser quantity of key aroma compounds with fatty and cheese aromas [19].

**Figure 2:**
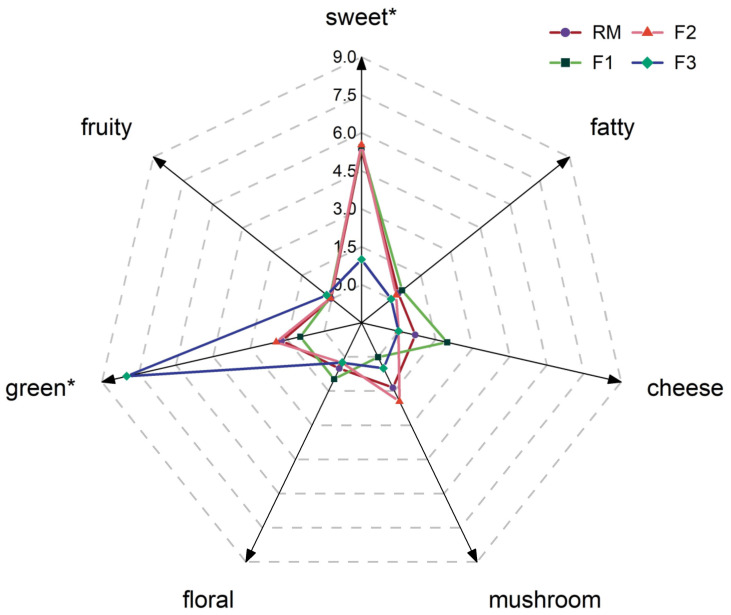
Aroma profile of three cultivars of *F. filiformis* and aroma recombinant model. * *p* ≤ 0.05. RM: recombinant model.

To identify the most robust aroma in the yellow cultivars, significant odorants (*p* ≤ 0.05) in omission tests were selected and added to the matrix to create a reconstitution model for F2. In addition, a sensory comparison was conducted between the aroma profiles of the three mushroom cultivars. Based on Figure 2, the sensory panel compared the seven aroma attributes in the recombination model with the three cultivars and scored these attributes (on a scale of 10). First, there was a significant difference (*p* ≤ 0.05) in the sweet and green notes among the three cultivars, which is consistent with the differences in their key aroma compounds. At the same time, it suggested that aroma compounds were the origin of aroma attributes. This could be because the green note in *F. filiformis* primarily came from volatile aldehydes and alcohols, which were relatively challenging to extract [39]. Moreover, the complexity of aroma formation (the synergy between aroma compounds) and the possible existence of undetectable substances could also result in variations in aroma traits [14].

Then, the calculation of OAVs and the AEDA were methods used to assess the contribution of compounds to the overall aroma. These methods incorporated human perception, specifically odor thresholds and ODP olfactory ports, to determine the contribution of each compound. However, further research is necessary to fully understand the relationships between these two factors. For example, the FD factors of these compounds showed a positive correlation with the OAV, such as 3-methylbutyl octanoate, 3-octanone, octyl acetate, and 3-octanol. In contrast, the FD factors of these compounds were relatively high; however, their OAVs were less than one. Consequently, the recreation of the initial aroma characteristics in cultivars using recombination models proved to be a demanding task, requiring further investigation in subsequent research [36]. However, considering the high similarity between the recombination model and the yellow variety (F2), it can be deemed a successful recombination model for yellow *F. filiformis* in this study.

### 3.5. Aroma Attributes’ Correlation with Aroma-Active Compounds Using Partial Least-Squares Regression (PLSR)

**Figure 3:**
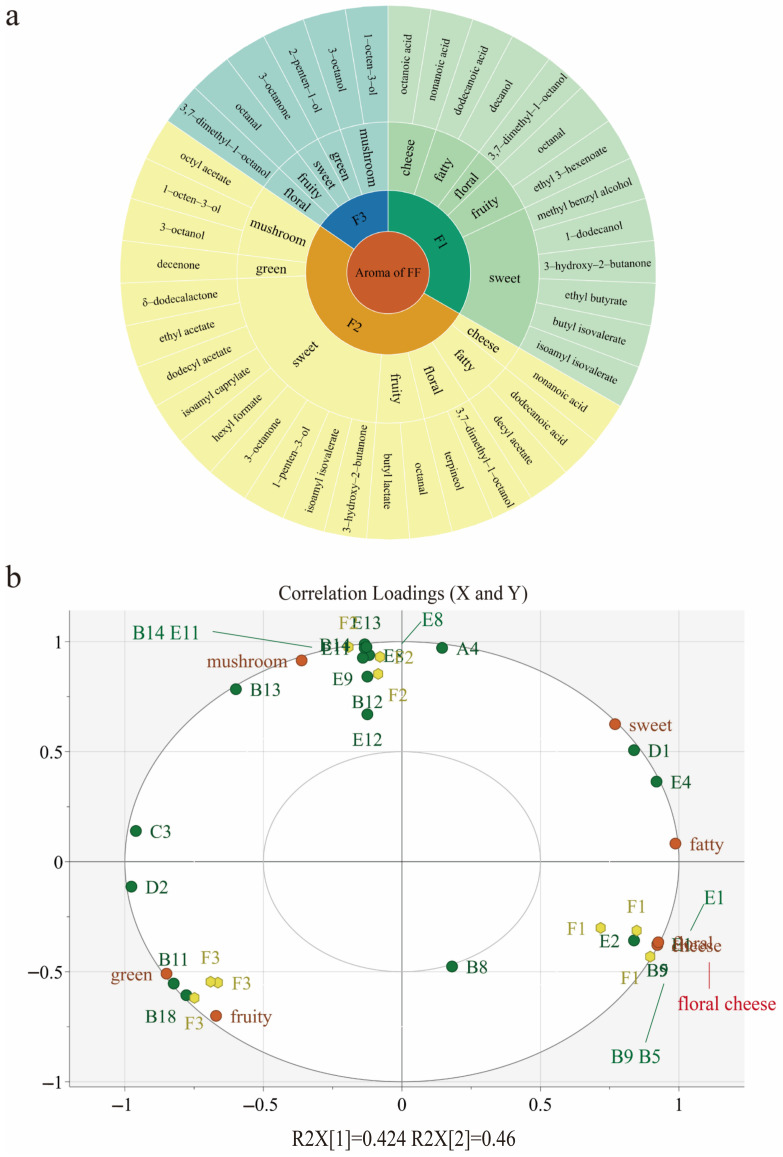
(**a**) Aroma wheel of key odorants and aroma attributes from three *F. filiformis* (FF) cultivars; (**b**) correlation loading plot for aroma-active compounds (X-matrix) and aroma attributes from *F. filiformis* (Y-matrix).

Using validated key aroma compounds obtained from the recombination experiments, a PLSR model was utilized to determine the relationships between aroma characteristics and key volatiles (Figure 3b). The cultivars were tested thrice, and the outcomes displayed reliable repeatability with good clustering. The load chart showed two ellipses, one representing 50% and the other 100% of the explanatory variable [19]. All the aroma attributes fell between these two ellipses, and the correlation coefficients of R^2^, X_1_ = 0.424, and X_2_ = 0.46, with a total of 0.884, indicated that the PLSR model was effective in explaining the correlation between sensory evaluation and compounds. Upon analysis of the correlation chart, it was revealed that F3 was closely correlated with green and fruit notes. The sweet attribute was distributed between F1 and F2, being furthest from F3. This observation leads to the hypothesis of a correlation between the sweet note and the respective cultivars (F1 and F2), which is consistent with previous findings (Figure 2).

This study focused on identifying the key aroma compounds in three types of *F. filiformis* and their seven aroma attributes. Based on the results, an aroma wheel was created to describe the aroma characteristics of F1, F2, and F3 in *F. filiformis* (Figure 3a). As a result, the research concluded that in F1, five odorants were found to be the main contributors to sweet notes (B9, D1, E1, E2, and E4). The compounds mentioned were observed to be closer to F1 on the PLSR plot. Notably, 3-hydroxy-2-butanone and isopentyl 3-methylbutanoate showed a significant correlation with sweet notes. 3-hydroxy-2-butanone, which has been identified as a key aroma compound in *F. filiformis*, was linked to the quality of “creamy” in previous studies [40]. In addition, nonanoic acid contributed to a cheese-like odor, whereas octanal played a vital role in fruity notes. Octanal is considered a key aroma compound in many edible fungi and is mainly generated through the oxidation or photochemical degradation of toluene or other hydrocarbons [9,33].

Secondly, seven odorants were identified in F2 as having the greatest effect on sweet notes (B12, D1, E4, D5, E9, E12, and D2). Obviously, B12, D5, E9, and E12 showed a strong correlation with F2, according to the correlation map. Furthermore, 3-octanol, 1-octen-3-ol, and 1-octen-3-ol were characteristic compounds that contributed to the mushroom note, which is consistent with studies on other edible fungi [9]. Terpineol was considered a potential contributor to floral notes.

Furthermore, the aroma wheel of F3 included sweet, floral, green, and fruity notes, attributed to by key components like 3-octanone, 2-penten-1-ol, and octanal, respectively. On the other hand, 2-penten-1-ol was closely associated with green notes in the PLSR plot, indicating a strong correlation. The mushroom note in F3 was derived from compounds like 3-octanol and 1-octen-3-ol. In this study, it was found that certain compounds played a crucial role in the aroma present in edible mushrooms like *F. filiformis*. These compounds not only contributed to the typical mushroom note but also offered various other aromas, such as fruity and sweet notes, which were crucial for analyzing the aroma in *F. filiformis*. While this study provided a comprehensive analysis and quantification of the volatile components in *F. filiformis*, several limitations should be acknowledged. This study did not explore the enzymes and corresponding genes involved in the synthesis of volatile compounds in *F. filiformis*, warranting future research to elucidate the aroma synthesis mechanisms and contribute to breeding initiatives aimed at enhancing aroma robustness.

## 4. Conclusions

In this study, the volatiles from three types of *F. filiformis* were extracted using both HS-SPME and SAFE methods and identified through GC-MS-O and AEDA. Based on their high FD values, volatile compounds with a high aroma contribution were selected for external standard quantification. Among all the quantitative compounds, 28 odorants were determined to be the main aroma volatiles due to their high OAVs (OAVs ≥ 1). Moreover, 20 key aroma compounds were identified through aroma recombination and omission experiments as further verification. Finally, the correlation between key odorants and aroma characteristics was evaluated by PLSR, and aroma wheels were plotted for the three types of *F. filiformis*. In conclusion, the dominant aroma in the yellow cultivar is sweet, with 3-hydroxy-2-butanone and isopentyl 3-methylbutanoate as the common main contributors in F1 and F2. In contrast, the characteristic aroma of the white cultivar is green, with 2-penten-1-ol being the major contributor. In particular, isopentyl 3-methylbutanoate and 2-penten-1-ol were identified as key aroma compounds in *F. filiformis* for the first time. This work offers significant insights into elucidating the aroma variation between yellow and white strains. And this research will also help improve the current situation regarding the preference of consumers for the aroma in yellow cultivars and the lack of diversification in *F. filiformis* cultivars. Therefore, further research is required to explore the enzymes and corresponding genes regulating the synthesis of aroma compounds in *F. filiformis* for breeding initiatives with both high yields and a robust aroma.

## Figures and Tables

**Table 1:** Identification of aroma-active compounds in *F. filiformis*.

					FD ^c^			
No.		Name	RI/KI ^a^	OD ^b^	F1	F2	F3	IM ^d^
acids	A1	isobutyric acid	1318	sour, cheese	1			MS, O, RI
	A2	dodecanoic acid	1947	fatty, coconut	128	4		MS, O, RI
	A3	octanoic acid	2053	cheese, fat, grass	4			MS, O, RI
	A4	nonanoic acid	2157	cheese	512	1		MS, O, RI
alcohols	B1	3-methyl-1-butanol	1218	burnt, cocoa, floral, malt	1	512		MS, O, RI
	B2	dodecanol	1264	earthy, sweet, honey, coconut	2			MS, O, RI
	B3	2-ethyl-1-hexanol	1483	green, rose	2	2	1	MS, O, RI
	B4	2-heptanol	1575	citrus, earth, fried, mushroom	1	8		MS, O, RI
	B5	2-octanol	1599	fat, mushroom	1			MS, O, RI
	B6	2-nonanol	1618	cucumber, green	1			MS, O, RI
	B7	hexadecanol	1638	waxy, clean, floral, oily	1			MS, O, RI
	B8	3,7-dimethyl-1-octanol	1641	floral	256	8	1	MS, O, RI
	B9	methyl benzyl alcohol	2180	sweet, gardenia, floral	2			MS, O, RI
	B10	decanol	1385	fat, oil	16			MS, O, RI
	B11	3-octanol	1261	mushroom		512	1	MS, O, RI
	B12	1-penten-3-ol	1333	butter, sweet		64		MS, O, RI
	B13	1-octen-3-ol	1410	earth, fat, mushroom		4	1	MS, O, RI
	B14	terpineol	1867	floral		128		MS, O, RI
	B15	2-pentanol	1095	oil, green			1	MS, O, RI
	B16	2,4-decadien-1-ol	1225	fatty, citrus			2	MS, O, RI
	B17	1-octanol	1403	bitter almond, fat, floral			1	MS, O, RI
	B18	2-penten-1-ol	1333	green			32	MS, O, RI
aldehydes	C1	2-dodecenal	1293	fruit, citrus	1			MS, O, RI
	C2	3,7-dimethyl-2,6-octadienal	1339	lemon	8			MS, O, RI
	C3	octanal	1347	citrus, fat, green, oil	8	16	1	MS, O, RI
	C4	nonanal	1399	fat, floral, green, lemon	2	1		MS, O, RI
	C5	2-undecenal	1245	citrus, orange peel		8	1	MS, O, RI
	C6	2-nonenal	1293	green, cucumber		1024		MS, O, RI
	C7	4-decenal	1738	citrus		1	1	MS, O, RI
ketones	D1	3-hydroxy-2-butanone	1583	sweet, buttery, creamy	2	1		MS, O, RI
	D2	3-octanone	1269	butter, herb, sweet, mushroom		512	4	MS, O, RI
	D3	1-hepten-3-one	1641	metallic		16		MS, O, RI
	D4	decenone	1692	fatty, green, fruity		1024		MS, O, RI
	D5	*δ*-dodecalactone	2146	fruit, sweet, peach, coconut		1		MS, O, RI
esters	E1	ethyl butyrate	1188	apple, sweet, cheese, pineapple	1			MS, O, RI
	E2	butyl 3-methylbutanoate	1685	fruity, apple, sweet	64			MS, O, RI
	E3	ethyl 3-hexenoate	1841	fruity, pineapple, green	8			MS, O, RI
	E4	isopentyl 3-methylbutanoate	1855	sweet, fruity, apple	64	256		MS, O, RI
	E5	butyl pentanoate	1888	sweet, fruity, pineapple	256			MS, O, RI
	E6	heptyl methanoate	1222	green, floral, apple		1		MS, O, RI
	E7	hexyl methanoate	1403	apple, banana, sweet		1		MS, O, RI
	E8	octyl acetate	1458	green, earthy, mushroom		512		MS, O, RI
	E9	3-methylbutyl octanoate	1829	sweet, fruity, pineapple		1024		MS, O, RI
	E10	dodecyl acetate	1907	sweet, waxy		4		MS, O, RI
	E11	decyl acetate	1929	oil, orange		128		MS, O, RI
	E12	ethyl acetate	2114	sweet, pineapple		8		MS, O, RI
	E13	isopentyl isobutyrate	1655	fruity, green, grape			2	MS, O, RI
	E14	ethyl oleate	2225	fatty, milky			8	MS, O, RI
	E15	1-octen-3-ol butyrate	1874	fruity, floral			8	MS, O, RI
others	F1	limonene	1222	citrus, mint	1			MS, O, RI
	F2	2-methylpyrazine	1277	cocoa, green	1			MS, O, RI
	F3	3-methylpyrazine	2013	nut	1			MS, O, RI
	F4	2-isopropyl pyrazine	2181	minty, green, nutty, honey		4		MS, O, RI

^a^ retention index (Kovats index) of odorants on HP-INNOWAX column. ^b^ odor descriptors from the olfactory detection port ODP-4. ^c^ FD factors determined on the HP-INNOWAX column. ^d^ identification method: MS means identification by comparison with the NIST 23 mass spectral database; O means confirmed by aroma descriptors; and RI means confirmed by comparison of the retention index with reference standards (https://webbook.nist.gov/, accessed on 7 August 2022).

**Table 2:** Concentration, odor threshold, and OAV of key aroma-active odorants in three cultivars of *F. filiformis*.

		Concentration (mg/kg) ^c^					OAV ^f^		
No.	Name	F1	F2	F3	QI ^d^	OT ^e^	F1	F2	F3
A2	dodecanoic acid	0.0447 ± 0.0136 ^b^	0.1446 ± 0.0428 ^a^		43, 60, 73	11.2681	4	13	
A3	octanoic acid	0.0207 ± 0.0095			60, 73	0.19	109		
A4	nonanoic acid	0.0832 ± 0.0070 ^b^	0.2966 ± 0.0435 ^a^		57, 60, 73	5.8471	14	51	
B9	methyl benzyl alcohol	0.5072 ± 0.0756			77, 79, 107	0.4074	>1000		
B10	decanol	0.0675 ± 0.0008			55, 70	2.5918	26		
B11	3-octanol		0.0604 ± 0.0266 ^b^	0.7607 ± 0.1035 ^a^	55, 59, 83	0.1709		354	>1000
B12	1-penten-3-ol		0.0189 ± 0.0129		57	0.1578		120	
B13	1-octen-3-ol		0.2851 ± 0.0189 ^a^	0.1421 ± 0.0357 ^b^	43, 57	0.0625		>1000	>1000
B14	terpineol		0.1339 ± 0.0048		59, 93, 121	0.7509		178	
B2	1-dodecanol	0.0052 ± 0.0069			43, 55, 69	3.4348	2		
B18	2-penten-1-ol			3.4681 ± 0.6225	57	0.72			>1000
B8	3,7-dimethyl-1-octanol	0.5577 ± 0.0211 ^a^	0.5355 ± 0.0262 ^a^	0.5531 ± 0.0039 ^a^	41, 55, 56	0.0009	>1000	>1000	>1000
C3	octanal	0.0259 ± 0.0006 ^b^	0.0317 ± 0.0007 ^a^	0.0336 ± 0.0014 ^a^	43, 44	0.0034	>1000	>1000	>1000
D1	3-hydroxy-2-butanone	27.9547 ± 4.9313 ^a^	24.4867 ± 3.6591 ^a^		43, 45	0.59	>1000	>1000	
D2	3-octanone		0.8568 ± 0.2728 ^b^	1.6570 ± 0.1777 ^a^	43, 57, 72	0.0330		>1000	>1000
D4	decenone		0.1801 ± 0.0148		43, 55	10.2799		18	
D5	*δ*-dodecalactone		0.6141 ± 0.0720		99	0.098		>1000	
E1	ethyl butyrate	0.0972 ± 0.0109			43, 71	0.0104	>1000		
E7	hexyl methanoate		0.0852 ± 0.0372		56	8.8135		10	
E8	octyl acetate		0.2401 ± 0.0665		43	0.1105		>1000	
E9	3-methylbutyl octanoate		2.0699 ± 0.8334		70, 127	0.07		>1000	
E10	dodecyl acetate		0.1956 ± 0.0039		43, 55	49.9471		4	
E11	decyl acetate		0.1208 ± 0.0254		43, 70	0.2903		416	
E12	ethyl acetate		0.0277 ± 0.0322		43	0.0194		>1000	
E2	butyl 3-methylbutanoate	0.0519 ± 0.0261			56, 57, 85	0.1786	290		
E3	ethyl 3-hexenoate	0.3146 ± 0.0596			29, 41, 69	103.7098	3		
E4	isopentyl 3-methylbutanoate	0.3177 ± 0.0357 ^a^	0.2264 ± 0.0270 ^b^		43, 70, 85	0.02	>1000	>1000	
E5	butyl pentanoate	0.0064 ± 0.0016			56, 57, 85	25.4167	<1		

^c^ external standard curve correction concentration; results were expressed as the mean value (*n* = 3). Values bearing different lowercase roman letters (^a^ and ^b^) were significantly different (*p* < 0.05). The actual concentration is 10^3^ of the value shown in the table for purposes of aesthetics. ^d^ quantification ions, selected for quantitation according to Huang et al. [36]. ^e^ odor threshold (mg/kg). ^f^ ratio of concentration to the threshold.

**Table 3:** Omission experiments of *F. filiformis* based on the complete aroma recombination model.

Test No.	Omitted Odorants	Difference in Odor	Number of Correct Answers ^a^
1	sweet note compounds	less sweet	9 **
1-1	1-penten-3-ol	less butter, less sweet	5 *
1-2	3-hydroxy-2-butanone	less creamy, less sweet	9 **
1-3	isopentyl 3-methylbutanoate	less sweet, less fruity	8 **
1-4	*δ*-dodecalactone	less fruity, less sweet	7 **
1-5	ethyl butyrate	less cheese, less sweet	8 **
1-6	butyl 3-methylbutanoate	less sweet, less apple-like	5 *
1-7	hexyl methanoate	nd ^b^	2
1-8	3-methylbutyl octanoate	less sweet, less fruity	9 **
1-9	ethyl acetate	less sweet	6 *
1-10	methyl benzyl alcohol	less floral, less sweet	6 *
1-11	1-dodecanol	nd ^b^	1
1-12	dodecyl acetate	nd ^b^	1
1-13	3-octanone	less sweet, less herb	9 **
2	fatty note compounds	mildly less fatty	6 *
2-1	dodecanoic acid	nd ^b^	2
2-2	decanol	nd ^b^	3
2-3	decyl acetate	less fatty	5 *
3	cheese note compounds	decreased acidic	4 *
3-1	octanoic acid	nd ^b^	1
3-2	nonanoic acid	mildly less acidic	4 *
4	mushroom note compounds	less mushroom-like	9 **
4-1	3-octanol	less earthy	5 *
4-2	1-octen-3-ol	less mushroom	7 **
4-3	2-octanol	less mushroom	9 **
4-4	octyl acetate	less mushroom	6 *
5	floral note compounds	slightly less floral	9 **
5-1	terpineol	decreased floral	5 *
5-2	3,7-dimethyl-1-octanol	decreased floral	9 **
6	green note compounds	less green	8 **
6-1	decenone	nd ^b^	2
6-2	2-penten-1-ol	less green	7 **
7	fruity note compounds	less fruity, less sweet	8 **
7-1	ethyl 3-hexenoate	nd ^b^	1
7-2	octanal	less citrus	8 **

^a^ The number of panelists who distinguished the aroma difference using a triangle test. Ten panelists were invited to participate in the aroma omission experiment. Values bearing different symbols (* and **) were significantly different: ** *p* ≤ 0.01; * *p* ≤ 0.05. ^b^ nd means not detectable.

## Data Availability

Data will be made available upon request. Email address: fengtao@sit.edu.cn.

## Supplementary Materials

The following supporting information can be downloaded at https://www.mdpi.com/article/10.3390/foods13050684/s1: Table S1. Three cultivars of *F. filiformis*; Table S2. Chemical standards; Table S3. Highest presented aroma compound concentrations for OT determination; Table S4. Standard curves for aroma-active compounds in *F. filiformis*; Table S5. Supplementary information about aroma-active compounds in *F. filiformis.*
