# Supplementary material for: Variations in Key Aroma Compounds and Aroma Profiles in Yellow and White Cultivars of Flammulina filiformis Based on Gas Chromatography–Mass Spectrometry–Olfactometry, Aroma Recombination, and Omission Experiments Coupled with Odor Threshold Concentrations
Source: Foods. 2024 Feb 23;13(5):684. doi: 10.3390/foods13050684 (PMC10931242; doi:10.3390/foods13050684)
Supplement: Supplementary file 1 [file foods-13-00684-s001.zip › foods-2862333-supplementary.pdf]

Supplementary data

Table captions:

Table S1. Three Samples of *F. filiformis*

Table S2. Chemical Standards

Table S3. Highest presented aroma compound concentrations for OT determination

Table S4. Standard Curves for Aroma-Active Compounds in *F. filiformis*

Table S5. Supplementary information of Aroma-Active Compounds in *F. filiformis.*

Table S1. Three samples of *F. filiformis*

Strain	Name	Number of shoots	Single bottle yield/g	Stem length/cm	Stem diameter/mm	Cap diameter/mm
No.61524	F1	348	267.32	13.22	3.66	7.29
No.61532	F2	674	354.00	17.96	3.18	8.52
No.61490	F3	965	468.18	17.35	3.56	8.38

Table S2. Chemical Standards

Boer (Shanghai, China)		Adamas (Shanghai, China)		Sigma-Aldrich (Shanghai, China)		Sinopharm (Shanghai, China)	
butyl 3-methylbutanoate	97%+	hexyl methanoate	>98.0%	acetone	AR	dichloromethane	AR
3,7-dimethyl-1-octanol	98%	octanal	98%	1,2-dichlorobenzene	AR	anhydrous sodium sulfate	AR
1-penten-3-ol	98%	methyl benzyl alcohol	≥98%	C7-C30 n-alkanes (solvent: hexane)	AR	sodium chloride	AR
1-octen-3-ol	98%	δ-dodecalactone	99%				
ethyl 3-hexenoate	98%	butyl pentanoate	98%				
ethyl acetate	99.7%+	3-octanol	>98%				
decanol	99.5%	dodecanoic acid	99%				
3-octanone	98%	octanoic acid	99%				
ethyl butyrate	99.5%	dodecyl acetate	95%				
terpineol	98%+	dodecanol	>99%				
		decyl acetate	≥99%				
		nonanoic acid	≥98%				
		octyl acetate	93%				
		decenone	≥98%				
		isopentyl 3-methylbutanoate	>97%				

3-hydroxy-2-butanone	98%
3-methylbutyl octanoate	≥95%
2-penten-1-ol	≥95%

Table S3. Highest presented aroma compound concentrations for OT determination

No.	Compounds	maximum concentration (mg/kg)
A2	dodecanoic acid	270
A4	nonanoic acid	124
B9	methyl benzyl alcohol	13
B10	decanol	21
B11	3-octanol	2
B12	1-penten-3-ol	10
B13	1-octen-3-ol	1
B14	terpineol	32
B2	dodecanol	67
B8	3,7-dimethyl-1-octanol	0.004
D2	3-octanone	1
D4	decenone	249
E1	ethyl butyrate	0.2
E7	hexyl methanoate	598
E8	octyl acetate	1
E10	dodecyl acetate	134
E11	decyl acetate	6

E12	ethyl acetate	0.1
E2	butyl 3-methylbutanoate	2
E3	ethyl 3-hexenoate	242
E5	butyl pentanoate	314

Table S4. Standard Curves for Aroma-Active Compounds in *F. filiformis*

No.	compounds	Standard curve	R^2	LOD (μg/kg)	LOQ (μg/kg)
A2	dodecanoic acid	y=0.9133x+0.1042	0.9505	0.0055	0.0182
A3	octanoic acid	y=1.8108x+0.1237	0.9535	0.0084	0.0280
A4	nonanoic acid	y=0.4687x+0.3528	0.9374	0.0195	0.0648
B9	methyl benzyl alcohol	y=0.7178x+0.0612	0.9891	0.0588	0.1960
B10	decanol	y=1.1604x+0.1086	0.9441	0.0230	0.0767
B11	3-octanol	y=0.4477x+0.0684	0.9830	0.0059	0.0198
B12	1-penten-3-ol	y=0.1936x+0.0362	0.9703	0.0344	0.1146
B13	1-octen-3-ol	y=0.3245x+0.1569	0.9865	0.0243	0.0808
B14	terpineol	y=1.2673x+0.2444	0.9379	0.0080	0.0268
B2	dodecanol	y=0.6596x+0.0426	0.9999	0.0314	0.1048
B18	2-penten-1-ol	y=0.0098x+0.0104	0.9988	0.0048	0.0158
B8	3,7-dimethyl-1-octanol	y=1.1345x+0.8208	0.9273	0.0070	0.0235
C3	octanal	y=1.9613x-0.0197	0.9994	0.3072	1.0240
D1	3-hydroxy-2-butanone	y=0.0089x+0.0306	0.9744	0.0137	0.0456
D2	3-octanone	y=0.2662x+0.5175	0.9253	0.0032	0.0106
D4	decenone	y=0.4744x+0.1512	0.9834	0.0037	0.0123

D5	δ-dodecalactone	y=0.1609x+0.0320	0.9690	0.0616	0.2054
E1	ethyl butyrate	y=0.1737x+0.0041	0.9726	0.0039	0.0130
E7	hexyl methanoate	y=0.2441x+0.0560	0.9551	0.0370	0.1233
E8	octyl acetate	y=0.3019x+0.1161	0.9541	0.0067	0.0222
E9	3-methylbutyl octanoate	y=0.0370x+0.2612	0.9494	0.0395	0.1315
E10	dodecyl acetate	y=1.4696x-0.2433	0.9812	0.0690	0.2299
E11	decyl acetate	y=0.3015x+0.1037	0.9753	0.0109	0.0364
E12	ethyl acetate	y=0.6899x+0.1951	0.9715	0.0044	0.0146
E2	butyl 3-methylbutanoate	y=0.5450x+0.1478	0.9611	0.0076	0.0252
E3	ethyl 3-hexenoate	y=0.3629x+0.0229	0.9848	0.0340	0.1132
E4	isopentyl 3-methylbutanoate	y=0.9009x-0.0497	0.9983	0.0233	0.0778
E5	butyl pentanoate	y=2.2070x+0.0601	0.9913	0.0508	0.1694

Table S5. Supplementary information of Aroma-Active Compounds in *F. filiformis*

no.		name	chemical formula	Molecular mass	Quantification Ions
acids	A1	isobutyric acid	C4H8O2	88.10512	43,41
	A2	dodecanoic acid	C12H24O2	200.31776	43,60,73
	A3	octanoic acid	C8H16O2	144.21144	60,73
	A4	nonanoic acid	C9H18O2	158.23802	57,60,73
alcohols	B1	3-methyl-1-butanol	C5H12O	88.15	55,42,43
	B2	dodecanol	C12H26O	186.339	43,55,69
	B3	2-ethyl-1-hexanol	C8H18O	130.231	57
	B4	2-heptanol	C7H16O	116.2	45
	B5	2-octanol	C8H18O	130.23	45
	B6	2-nonanol	C9H20O	144.258	45

	B7	hexadecanol	C16H34O	242.447	55,69
	B8	3,7-dimethyl-1-octanol	C10H22O	158.28100	41,55,56
	B9	methyl benzyl alcohol	C8H10O	122.1644	77,79,107
	B10	decanol	C10H22O	158.285	55,70
	B11	3-octanol	C8H18O	130.23	55,59,83
	B12	1-penten-3-ol	C5H10O	86.13230	57
	B13	1-octen-3-ol	C8H16O	128.21	43,57
	B14	terpineol	C10H18O	154.24900	59,93,121
	B15	2-pentanol	C5H12O	88.14818	45
	B16	2,4-decadien-1-ol	C10H18O	154.24900	41
	B17	1-octanol	C8H18O	130.22792	55,56
	B18	2-penten-1-ol	C5H10O	86.134	57
aldehydes	C1	2-dodecenal	C12H22O	182.30200	41,43
	C2	3,7-dimethyl-2,6-octadienal	C10H16O	152.237	41,69
	C3	octanal	C8H16O	128.215	43,44
	C4	nonanal	C9H18O	142.23862	57,41,43
	C5	2-undecenal	C11H20O	168.28	41,70
	C6	2-nonenal	C9H16O	140.22	41,43
	C7	4-decenal	C10H18O	154.253	41
ketones	D1	3-hydroxy-2-butanone	C4H8O2	88.10512	43,45
	D2	3-octanone	C8H16O	128.215	43,57,72
	D3	1-hepten-3-one	C7H12O	112.172	55,70,27
	D4	decenone	C10H18O	154.253	43,55
	D5	δ-dodecalactone	C12H22O2	198.306	99
esters	E1	ethyl butyrate	C6H12O2	116.15828	43,71
	E2	butyl 3-methylbutanoate	C9H18O2	158.23802	56,57,85

	E3	ethyl 3-hexenoate	C8H14O2	142.198	29,41,69
	E4	isopentyl 3-methylbutanoate	C10H20O2	172.2646	43,70,85
	E5	butyl pentanoate	C9H18O2	158.23802	56,57,85
	E6	heptyl methanoate	C8H16O2	144.21	70,56,41
	E7	hexyl methanoate	C7H14O2	130.187	56
	E8	octyl acetate	C10H20O2	172.2646	43
	E9	3-methylbutyl octanoate	C13H26O2	214.34434	70,127
	E10	dodecyl acetate	C14H28O2	228.37092	43,55
	E11	decyl acetate	C12H24O2	200.322	43,70
	E12	ethyl acetate	C4H8O2	88.10512	43
	E14	isopentyl isobutyrate	C9H18O2	158.23802	43,71
	E15	ethyl oleate	C20H38O2	310.51452	43
	E16	1-octen-3-ol butyrate	C12H22O2	198.30188	43,54,71
others	F1	limonene	C10H16	136.23404	68,93
	F2	2-methylpyrazine	C5H6N2	94.117	94,67
	F3	3-methylpyrazine	C7H10N2	122.168	42,122
	F4	2-isopropyl pyrazine	C7H10N2	122.171	-
